# Primary Hemostasis in Chronic Liver Disease and Cirrhosis: What Did We Learn over the Past Decade?

**DOI:** 10.3390/ijms21093294

**Published:** 2020-05-06

**Authors:** Marie-Astrid van Dievoet, Stéphane Eeckhoudt, Xavier Stephenne

**Affiliations:** 1 Département des Laboratoires Cliniques, Cliniques universitaires Saint-Luc, 1200 Brussels, Belgium; Stephane.EECKHOUDT@LHUB-ULB.be; 2 Laboratoire d’Hépatologie Pédiatrique et Thérapie Cellulaire, Unité PEDI, Institut de Recherche Expérimentale et Clinique (IREC), Université Catholique de Louvain (UCLouvain), 1200 Brussels, Belgium; xavier.stephenne@uclouvain.be; 3 Service de Gastro-Entérologie et Hépatologie Pédiatrique, Département de Pédiatrie, Cliniques Universitaires Saint-Luc, 1200 Brussels, Belgium

**Keywords:** chronic liver disease, cirrhosis, primary hemostasis, platelet, platelet function, von Willebrand factor

## Abstract

Changes in primary hemostasis have been described in patients with chronic liver disease (CLD) and cirrhosis and are still subject to ongoing debate. Thrombocytopenia is common and multifactorial. Numerous studies also reported platelet dysfunction. In spite of these changes, primary hemostasis seems to be balanced. Patients with CLD and cirrhosis can suffer from both hemorrhagic and thrombotic complications. Variceal bleeding is the major hemorrhagic complication and is mainly determined by high portal pressure. Non portal hypertension-related bleeding due to hemostatic failure is uncommon. Thrombocytopenia can complicate management of invasive procedures in CLD patients. Recently, oral thrombopoietin agonists have been approved to raise platelets before invasive procedures. In this review we aim to bundle literature, published over the past decade, discussing primary hemostasis in CLD and cirrhosis including (1) platelet count and the role of thrombopoietin (TPO) agonists, (2) platelet function tests and markers of platelet activation, (3) von Willebrand factor and (4) global hemostasis tests.

## 1. Introduction

Chronic liver disease (CLD) was for a long time considered as a model of acquired hemorrhagic disease, reflected by, for example, prolonged prothrombin time. In the last 15 years, it has been acknowledged that CLD patients present complex alterations in the hemostatic system leading to so-called rebalanced hemostasis [1,2]. Patients with CLD and cirrhosis can suffer from both hemorrhagic and thrombotic complications. Regarding hemorrhage, a distinction is made between portal hypertension-related bleeding, spontaneous bleeding and provoked bleeding. Gastrointestinal (variceal) bleeding is common in CLD and cirrhosis and is due to the elevation in portal pressure giving rise to shunts between the portal and caval system. The risk of variceal hemorrhage is mainly determined by portal hypertension (hepatic venous pressure gradient > 12 mmHg), severity of liver disease, the size of varices and presence of red signs [3,4]. Variceal hemorrhage is not related to true hemostatic failure. The fragile balance recognized in cirrhotic patients can also tip towards thrombosis. Thrombotic complications encompass portal vein thrombosis (PVT) and venous thromboembolism. PVT is mainly caused by reduced portal flow (< 15 cm/s) [5]. In a recent meta-analysis, an increased venous thromboembolic risk was confirmed in cirrhotic patients with a prevalence of 3.7% [6]. Cirrhotic subjects were more at risk of deep venous thrombosis and pulmonary embolism compared to controls. A lower incidence of PVT, hepatic decompensation and an improved survival were reported in patients with advanced cirrhosis, randomized to prophylaxis with low molecular weight heparin, compared to standard of care [7]. Multiple authors have described a hypercoagulable state in cirrhotic patients [8,9,10,11,12]. Furthermore, liver transplantation without the need for blood products is feasible in this patient group [13].

Although rebalanced hemostasis is substantially studied for the coagulation cascade, alterations in primary hemostasis are not well understood. Variable thrombocytopenia is present in the majority of CLD patients [14]. This is in part counterbalanced by elevated von Willebrand factor (VWF) supporting enhanced platelets’ adhesion [15,16]. By their complex phenotype, platelets play a diverse and key role in hemostasis. However, their role in rebalanced hemostasis is not clear. In this review we will highlight current knowledge of primary hemostasis in CLD.

## 2. Chronic Liver Disease and Platelet Count

### 2.1. Studies on CLD and Platelet Count

Thrombocytopenia is common in patients with CLD. The prevalence depends on disease severity and the criteria used: 6% in chronic hepatitis patients versus 64% in cirrhotic patients [14]. A low platelet count is an indicator of more advanced disease and is associated with poorer prognosis [17,18]. In patients with chronic hepatitis C infections, immune thrombocytopenic purpura can also cause low platelet counts asymmetric to portal hypertension and hypersplenism. Platelets have been implicated in both CLD progression and liver regeneration. This topic is extensively reviewed elsewhere and will not be discussed here [19].

In a prospective study [17], the non-provoked bleeding rate was investigated in 280 patients with cirrhosis. Overall, 52 patients experienced a major or minor bleeding event over four years (5.45%/year). No association was observed between platelet count and bleeding. Two studies addressing variceal bleeding found opposite results regarding the association between platelet count and bleeding [20,21]. It is, however, important to stress that platelet count may merely reflect the degree of portal hypertension responsible for variceal hemorrhage [22]. Low platelet count was identified as a risk factor for major bleeding in a retrospective study of cirrhotic patients admitted to intensive care [23].

By possibly increasing the risk of bleeding, severe thrombocytopenia can complicate the management of patients with CLD undergoing invasive procedures. A threshold of 50 × 10^9^/L is often used for platelet transfusion to mitigate the risk of procedure-related bleeding [24,25,26]. This platelet transfusion cut-off is also based on a paper by Tripodi and colleagues [27]. They demonstrated that a platelet count of 56 × 10^9^/L was necessary for normal thrombin generation in platelet-rich plasma. In an article by Giannini et al [28], bleeding related to invasive procedures was more frequent in cirrhotic patients with severe thrombopenia (PLT < 75 × 10^9^/L, 10 out of 32 with procedure-related bleeding) compared to patients with moderate thrombocytopenia (75 × 10^9^/L< PLT< 150 × 10^9^/L, 0 out of 19 with procedure-related bleeding). Similarly, platelet count was strongly correlated with serious bleeding in patients with the hepatitis C virus (HCV) related to advanced CLD undergoing a liver biopsy. The bleeding risk increased significantly with platelet counts < 60 × 10^9^/L [26]. However, patients that experienced bleeding had more advanced liver disease, so it is not clear if platelet count caused the bleeding or was a manifestation of advanced liver disease (ie. bleeding caused by elevated liver stiffness, presence of ascites). The association of severe thrombocytopenia and post-procedural bleeding was also reported in a recent study on 874 cirrhotic patients [25]. In contrast to the previous findings, no post-procedural bleeding episode was reported in cirrhotic patients with a platelet count < 50 × 10^9^/L scheduled for an invasive procedure [29]. In a report of 500 orthotopic liver transplantations, no blood products were transfused in 79.6 % of patients [13]. A study addressing platelet transfusion did report a significant increase in platelet count, but did so without normalizing global hemostasis tests [30].

As an alternative to platelet transfusion, oral thrombopoietin (TPO) agonists, avatrombopag and lusutrombopag, were FDA approved in adult patients with CLD and a platelet count < 50 × 10^9^/L before an invasive procedure [24].

Different authors already reviewed the mechanisms of thrombocytopenia in CLD: splenic sequestration, decreased platelet production and increased platelet breakdown are involved [18,31,32,33]. Only TPO will be further discussed here due to the recent approval of TPO agonists. This does not imply that this is the most important mechanism for thrombocytopenia in CLD.

### 2.2. Thrombopoietin

Thrombopoietin (TPO), the humoral basis of thrombopoiesis, was revealed in 1994. [34]. TPO is a hematopoietic growth factor, produced primarily by the liver, which binds the c-MPL (myeloproliferative leukemia) receptor on megakaryocytes, platelets and most other hematopoietic precursor cells. In liver-healthy thrombocytopenic patients, TPO levels rise due to decreased degradation of TPO by platelets. Stimulation of megakaryocytes and an increase in platelet production ensues. However, in cirrhosis-associated thrombocytopenia, TPO levels may not rise sufficiently. Controversial reports exist regarding circulating TPO levels in cirrhotic patients. Some studies report low levels [35,36,37] while others report normal levels of TPO [18,38,39,40]. According to the prevailing theory of TPO regulation by “c-MPL mass”, circulating TPO levels, being the net effect of both production and clearance, do not reflect TPO production alone. The measurement of TPO mRNA expression in the liver better reflects TPO production and is decreased in patients with cirrhosis in most reports [41,42]. Currently, four TPO agonists exist: romiplostim (Nplate®; Amgen Inc., Thousand Oaks, CA, USA), eltrombopag (Revolade®; Promacta®; Novartis Pharma; Basel; Switzerland) avatrombopag (Doptelet®; Dova Pharmaceuticals; Durham, NC, USA) and lusutrombopag (Mulpleo®; Mulpleta®; Shionogi, Japan). Eltrombopag is not recommended as an alternative for platelet transfusion for patients with CLD due to thrombotic complications during the ELEVATE clinical trial [43]. Romiplostim is approved as second line therapy for chronic immune thrombocytopenic purpura, but not in CLD. Avatrombopag and lusutrombopag were approved during the ADAPT-1/2 and L-PLUS 1/2 trials, respectively [44,45,46]. Approximatly 70% of patients responded to their TPO agonist treatment. This means that 30% of patients are non-responders. An important advantage of TPO agonists is the avoidance of platelet transfusion and thereby not exposing patients to possible immunological or infectious risks as well as transfusion reactions and volume overload. It is important to know that TPO agonists take about ten days to elevate platelet count.

## 3. Chronic Liver Disease and Platelet Function

A variety of tests exist to assess platelet function. Some of them are extensively used, whereas others are confined to specialized or research laboratories. In vivo bleeding time was developed first but has become obsolete due to its invasive nature. The platelet function analyzer (PFA-100/200, Siemens Healthineers, Erlangen, Germany), a widespread and easy to perform assay, reflects high shear adhesion and aggregation during formation of a platelet plug. Platelet aggregation can be evaluated by light transmission aggregometry or whole blood aggregometry. Platelet glycoproteins and activation status (e.g., alpha granule release) can be appreciated by flow cytometry. In research, microfluidic devices have been developed for real-time assessment of thrombus formation in whole blood in response to a range of agonists. Soluble platelet release markers (e.g., soluble P-selectin and soluble CD40 ligand), also mainly used in research, investigate platelet activation state. PlateletMapping TEG technology employs specific platelet agonists but is mainly used for monitoring antiplatelet therapy [47].

About a decade ago, platelet function in CLD and cirrhosis was very thoroughly reviewed by Witters et al and Violi et al [11,33]. In our review, the focus will lie on more recent papers investigating this topic. Table 1 displays a chronological overview of recent studies (last ten years) of platelet function in CLD patients.

### 3.1. Platelet Aggregation Tests

As mentioned above, platelet aggregation is mostly studied by light transmission aggregometry or whole blood aggregometry. The following studies reported decreased platelet aggregation in CLD patients. In patients with chronic HCV, platelet aggregation measured with whole blood aggregometry was lower in more advanced fibrosis compared to mild fibrosis and healthy controls [48]. However, when platelet aggregation was corrected for platelet count and expressed as “multiplate units”, aggregation was higher in patients with advanced fibrosis. This corrected parameter is not validated and should be interpreted with caution as the significance and mechanisms are not clear. Preoperatively, Jüttner et al [49] demonstrated decreased platelet aggregation in cirrhotic patients of mixed etiology after adjustment for platelet count. Cholestatic patients had significantly higher maximal aggregation in comparison to non-cholestatic cirrhotic patients. They hypothesize a better preservation of parenchymal function in the former group. Wosiewicz et al [50] reported decreased platelet aggregation in cirrhotic patients with or without PVT awaiting liver transplantation. Curiously, patients with PVT had decreased platelet aggregation compared to patients without PVT (platelet count was similar between groups). In a pediatric cohort of decompensated cirrhotic children, we also highlighted decreased platelet aggregation [51]. Although not corrected for platelet count, aggregation was lower in children presenting with upper gastrointestinal bleeding. Eyraud et al [52] demonstrated improved platelet aggregation one month after liver transplantation compared to pre-transplant values. In a study by Vinholt et al [53], flow cytometry-based aggregation was significantly lower in cirrhotic patients after stimulation with platelet agonists. The independence of platelet count is an advantage of this technique. No platelet hyperaggregability was revealed after testing different agonist concentrations. In contrast to these findings, Raparelli et al [54] demonstrated enhanced platelet aggregation in cirrhotic patients after stimulation with subthreshold concentrations of platelet agonists. This difference in aggregation between patients and healthy controls was not detected with threshold concentrations of agonist. The addition of Toll-like receptor 4 binding protein, an inhibitor of the lipopolysaccharide acid (LPS) receptor, inhibited platelet aggregation only in cirrhotic patients. This study concluded that high circulating levels of LPS, translocated in the systemic circulation, may be the cause of enhanced platelet activation in cirrhotic patients.

### 3.2. Platelet Activation State by Flow Cytometry

Decreased platelet activatability assessed with P-selectin (= CD62P, alpha granule release), by flow cytometry, was seen in viral, non-alcoholic steatohepatitis, alcoholic steatohepatitis and cholestatic cirrhosis compared to controls [49,53,55,56]. At baseline, without stimulation, no difference in P-selectin expression between patients and controls was seen across different etiologies [53,55,56,57]. In line with these results, no differences in platelet activatability were seen before and after liver transplantation in cirrhotic patients [52]. Alcoholic steatohepatitis cirrhosis patients had lower percentages of platelets positive for CD63 (lysosomal marker) and activated GPIIb/IIIa after agonist stimulation compared to healthy controls [53], although some patients demonstrated normal platelet activatability. Platelet activation impairment was associated with severe liver disease (Child Turcotte Pugh B/C) and platelet count [53]. A lower response to stimulus rather than deficient GPIIb/IIIa was suggested since CD41 and CD61, GPIIb/IIIa subunits, were normally expressed. In contrast to these results, Sayed et al [58] and an older study by Panasiuk et al [59] demonstrated higher basal platelet activation in viral cirrhosis patients (P-selectin). As in the study by Raparelli et al [54] et al, they also reported increased platelet–monocyte complexes. Elevated platelet–leukocyte complexes were also confirmed by Støy et al’s study [60].

### 3.3. Soluble Release Markers

Increased platelet activation markers (soluble P selectin and/or soluble CD40 ligand), corrected or not for platelet count, in cirrhotic patients were demonstrated by several authors [8,54,55,56]. These levels were even more increased in decompensated patients and correlated well with serum LPS levels [54]. High oxidative stress and production of platelet isoprostanes were demonstrated to be related to higher soluble P selectin and soluble CD40 ligand levels [8,61]. In decompensated patients with a transjugular intrahepatic portosystemic shunt procedure soluble platelet activation markers, a soluble oxidative stress marker and LPS were elevated in the portal circulation. This suggested increased platelet activation with a possible contribution of oxidative stress and bacterial translocation [62]. The same authors found an association between elevated levels of thromboxane B2 in the portal circulation and portal hepatic venous pressure gradient. They concluded that thromboxane B2 is possibly associated with prothrombotic potential [63]. In the paper by Eyraud et al [52], soluble P selectin decreased after liver transplantation, suggesting a higher soluble P selectin/platelet count ratio in cirrhotic patients before transplantation. In compensated cirrhotic patients of mixed etiology, soluble glycoprotein VI was higher compared to healthy age-matched controls [64]. The authors interpreted this as an early onset marker of platelet activation. The majority of papers on soluble platelet release markers conclude a higher platelet activation state in CLD patients. However, these markers should be carefully interpreted since their elevation could also be a consequence of decreased clearance by the liver [65,66].

### 3.4. Platelet Function Analyzer and Studies under Flow Conditions

Under high shear flow conditions (5000–6000/s) platelets are sent through an aperture covered with collagen and epinephrine or adenosine diphosphate. Platelet adhesion and aggregation ensue. The closure time is recorded. The PFA is impacted by platelet count, VWF and hematocrit. It is insensitive to milder platelet disorders [67]. In patients with viral (HCV) cirrhosis PFA-100 closure times were prolonged compared to normal controls suggesting platelet hypofunction [68]. Patients with platelet count < 100 × 10^9^/L were excluded in this study. In a cohort of CLD patients awaiting liver transplantation, PFA-epinephrine was prolonged in 48.6% of patients [69]. As expected, platelet count and hematocrit predicted pathological PFA. VWF improved closure times partially. Another small study did not report added value of PFA in cirrhotic patients undergoing an invasive procedure. Only 16 patients were tested, all with thrombocytopenia (40 × 10^9^/L ± 20.7 × 10^9^/L). No details of PFA results were provided [70]. Lisman et al [15] studied platelet adhesion under flow conditions in a reconstituted blood model. Platelet count and hematocrit were adjusted in this experiment to 200 × 10^9^/L and 40%, respectively. Collagen and fibrinogen were used as surface activators and blood was passed through the chamber at a shear rate of 800/s and 300/s respectively. The capacity of cirrhotic platelets to adhere to the surface was identical to that of healthy volunteers.

## 4. Chronic Liver Disease and von Willebrand Factor

VWF exerts a pivotal role in primary hemostasis. Through its interaction with collagen and platelets, it localizes platelets to the site of vessel wall injury, allowing further adhesion. VWF plasma levels are known to be elevated in CLD [16,55,56,57,69,71,72,73,74,75,76,77]. The mechanism is not completely understood. Normally, VWF is mostly synthetized by the endothelial cells of large vessels. With liver injuries, VWF immunostaining also becomes positive in liver sinusoidal endothelial cells [78]. High VWF propeptide levels were reported in cirrhotic patients, which suggests ongoing acute endothelial damage [16,72,79]. Palyu et al demonstrated higher VWF propeptide levels in acutely decompensated cirrhotic patients. Decreased clearance by the liver should also be taken into account. VWF was demonstrated to be positively associated with more severe liver disease [16,74]. Although VWF seems to demonstrate a less efficient binding to platelet glycoprotein Ib, demonstrated by a reduced VWF activity to antigen ratio, the capacity to adhere platelets to a surface was preserved [16]. Interestingly, when platelets from healthy volunteers were mixed with cirrhotic plasma, platelet adhesion improved [16,72]. DDAVP (1-deamino, 8-D arginine-vasopressin) is known to increase VWF and factor VIII in hemophilia A and von Willebrand disease. In cirrhotic patients, no significant effect of an intravenous bolus of DDAVP on VWF plasma levels was seen. The authors hypothesized exhaustion of endothelial cells on the one hand and immediate intrahepatic consumption or endothelial attachment on the other hand [79]. These results were in contrast to the results of an earlier study reporting on the efficacy of intranasal DDAVP in cirrhotic patients undergoing dental extraction [80]. The design of the study was however questionable. A disintegrin-like and metalloproteinase with a thrombospondin type 1 motif, member 13 (ADAMTS13) cleaves to ultra-large VWF and is synthesized by the hepatic stellate cells in the liver [81,82]. Both reduced, normal and elevated ADAMTS13 levels have been reported [16,55,56,57,71,72,76,79]. Similarly, both presence and absence of high molecular weight VWF multimers in CLD patients were demonstrated [16,71,72]. No differences in VWF and ADAMTS13 levels were seen between different CLD etiologies [57].

## 5. Chronic Liver Disease and Global Hemostasis Tests

Global hemostasis tests like viscoelastic testing (VET) and the thrombin generation assay (platelet-rich plasma/whole blood) take into account the role of platelets and other blood components.

### 5.1. Use of Viscoelastic Assays and the Role of Platelets

Viscoelastic testing (VET) gives a more global picture of the hemostatic system compared to routine coagulation testing by better approaching in vivo clot formation. It is performed on whole blood taking into account platelets and other figurative elements. Two techniques are available: rotational thromboelastometry and thromboelastography. A limitation of VET is the lack of thrombomodulin, needed for full activation of protein C, and the insensitivity to VWF. VET parameters like maximal clot firmness (thromboelastometry) and maximal amplitude (thromboelastography) reflect platelet count but are largely independent of platelet function [83]. In stable cirrhosis, platelets are suggested to be the main determinant of clot formation [84]. VET is readily used in cardiothoracic surgery and bleeding trauma patients to guide transfusion. Similarly, in liver transplantation and invasive procedures in cirrhotic patients, VET can be used to guide blood product administration [85,86,87,88,89], although maximal clot firmness in FIBTEM conditions (addition of platelet inhibitor; thromboelastometry) is possibly less accurate in very low levels of fibrinogen (< 100 mg/dL) [90]. In CLD patients, outside the context of liver transplantation, VET parameters correlated well with platelet count, fibrinogen level and severity of liver disease [84,91,92]. Multiple authors reported both normal and hypocoagulable profiles in cirrhotic patients [91,92,93,94,95]. In a subset of patients with cholestatic cirrhosis, a hypercoagulable profile was shown [94]. No difference was, however, seen in viscoelastic parameters between stable cirrhotic patients with and without histories of bleeding or thrombosis [84,93]. Accordingly, thromboelastography parameters did not predict bleeding or thrombosis in cirrhotic patients with different etiologies [94]. A limitation of VET is the lack of a validated target level for cirrhotic patients in evaluation of their hemostatic system (for example in a pre-operative context) [24].

### 5.2. Thrombin Generation Assay

In the majority of centers, the thrombin generation assay is performed on platelet-poor plasma with or without thrombomodulin. Tripodi et al reported a minimum platelet count necessary for normal thrombin generation (56 × 10^9^/L) in platelet-rich plasma [27]. Very recently, Wan et al compared thrombin generation in whole blood with platelet-poor plasma in patients with cirrhosis and controls [96]. The advantage of a whole blood assay is the interaction with platelets, but also with leukocytes and erythrocytes. They reported lower peak thrombin generation in whole blood but preserved endogenous thrombin potential compared to controls. The authors hypothesize that this discordance between peak and endogenous thrombin potential could be explained by the more important impact of platelet and erythrocyte count on peak thrombin generation [97,98]. Interestingly, the anticoagulant effect of thrombomodulin was much less pronounced in the whole blood assay compared to platelet-poor plasma.

## 6. Conclusions

The study of primary hemostasis in CLD is complex and has not been extensively studied over the past decade. This is in part due to the technical difficulties met in studying platelets. Platelets are fragile and very easily activated in vitro. Platelet assays are not standardized: varying concentrations of agonists are, for example, used. Differences between studies can also be explained by the heterogeneity of the CLD population: different degrees of severity and different etiologies. Although no clear-cut platelet target is available, TPO agonists avatrombopag and lusutrombopag are a good alternative in CLD patients with severe thrombocytopenia before a high-risk invasive procedure. VWF plasma levels are increased in cirrhotic patients and support primary hemostasis in reconstituted blood models. The recent studies that did report in vivo platelet activation mostly analyzed soluble markers. An exception is the key study by Raparelli et al [54]. Overall, there is no clear consensus between papers regarding primary hemostasis in CLD. Ideally, platelet function tests approach in vivo platelet activation with minimal manipulation. In our opinion, microfluidic assays with correction for platelet count and hematocrit could be very interesting in future studies [99,100] and hopefully allow better management and risk assessment of these complicated patients to guide therapeutic decision making.

## Figures and Tables

**Table 1:** Published studies of platelet function in CLD patients over the past decade.

Reference	Population	Tests	Results
Jüttner et al., 2009 [49]	Tumor (*n* = 9) and cirrhosis (*n* = 25, mixed etiology)	LTA, mPs	decreased LTA in cirrhosis (cholestatic > non-cholestatic), decreased mPs in all patients
Sayed et al., 2010 [58]	cirrhosis (*n* = 60, viral)	platelet–monocyte complexes, mPs	increased
Basili et al., 2011 [8]	cirrhosis (*n* = 51, mixed etiology)	sNOX2dp, sPs, sCD40L, isoprostanes (urinary and platelet)	increased
Ghozlan et al., 2013 [68]	cirrhosis (*n* = 50, viral)	PFA-100 (COL/EPI)	prolonged CT
Basili 2014 [61]	cirrhosis (*n* = 51, mixed etiology)	serum polyunsaturated fatty acids, urinary isoprostanes, sNOXdp	higher n-6/n-3 polyunsaturated fatty acids ratio, correlation with elevated sNOXdp and isoprostanes
Alkozai et al., 2014 [55]	HCC (*n* = 22) and cirrhosis (*n* = 16, viral)	mPs (basal and stimulation), sPs	normal basal mPs, decreased stimulated mPs, increased sPs
Potze et al., 2016 [56]	NAFLD (*n* = 68, steatosis, NASH, NASH cirrhosis), ASH cirrhosis (*n* = 15)	mPs (basal and stimulation), sPs, PF4	normal basal mPs and PF4, decreased stimulated mPs in cirrhosis (NS), increased sPs (NS)
Egan et al., 2016 [64]	compensated cirrhosis (*n* = 46, mixed etiology)	soluble GPVI	increased
Wosiewicz et al., 2016 [50]	cirrhosis (*n* = 49, mixed etiology)	multiplate, zonuline, LPS (comparison presence/absence PVT)	decreased PA, NS difference zonuline, LPS between groups
Nielsen et al., 2017 [48]	chronic HCV (advanced fibrosis, *n* = 39; mild or no fibrosis, *n* = 43)	multiplate, TEG	decreased PA and increased PA/platelet count in advanced fibrosis, no difference in TEG
Vinholt et al., 2017 [53]	cirrhosis (*n* = 27, ASH)	96-well LTA, mPs, CD63, activated GPIIb/IIIa, FC platelet aggregation	decreased platelet aggregation/activatability
Raparelli et al., 2017 [54]	cirrhosis (*n* = 69, mixed etiology)	LTA, sPs, sCD40L, LPS, zonulin, platelet–monocyte complexes	increased sPs, sCD40L, LPS, zonulin, platelet–monocyte complexes, increased PA with STC (PLT adjusted)
Eyraud et al., 2018 [52]	HCC (*n* = 24) and cirrhosis (*n* = 26, mixed etiology)	LTA, mPs, platelet–leukocyte complexes, PDEV, sPs, sCD40L	decreased LTA pre-graft versus post-graft (D28, PLT adjusted ); mPs, sPs and sCD40L, platelet-leukocyte complexes unchanged, sPs slightly higher pre-graft
Stoy et al., 2018 [60]	Cirrhosis (*n* = 19, mixed etiology)	Platelet–leukocyte complexes	increased platelet-neutrophil complexes
Bonnet et al.,2019 [51]	decompensated cirrhotic children (*n* = 22, bililiary atresia, Alagille, other)	multiplate	decreased PA (adult RV, PLT not adjusted)
Queck et al., 2019 [62]	decompensated cirrhosis (*n* = 20 )	sPs, soluble GPVI, LPS, sNOXdp	increased in portal vein
Queck et al., 2019 [63]	decompensated cirrhosis (*n* = 89 )	TXB2 portal vein	association with PHPG increase after TIPS insertion
Rogalski et al., 2019 [21]	cirrhosis (*n* = 58, mixed etiology)	multiplate	comparable in patients with and without variceal bleeding

CT = closure time, COL = collagen, EPI = epinephrine, FC = flow cytometry, HCC= hepatocellular carcinoma, LPS = lipopolysaccharide acid, LTA = light transmission aggregometry, LT = liver transplantation, mPs = membrane P-selectin, NS = not significant, PA = platelet aggragation, PF4 = platelet factor 4, PFA = platelet function analyser, PHPG = portal hepatic venous pressure gradient, PLT = platelet, PVT = portal vein thrombosis, RV = refence values, sNOX2dp = soluble NADPH oxidase derived peptide, sPs = soluble P-selectin, STC = subtreshold concentrations, TIPS = transjugular intrahepatic portosystemic shunt.

## Abbreviations

ADAMTS13	A Disintegrin-like And Metalloproteinase with a ThromboSpondin type 1 motif, member 13
CLD	Chronic Liver Disease
c-MPL	c-MyeloProliferative Leukemia protein
LPS	LipoPolySaccharide acid
PFA	Platelet Function Analyzer
PVT	Portal Vein Thrombosis
TPO	Thrombopoietin
VET	Viscoelastic Testing
VWF	Von Willebrand Factor
